# Transfer Trauma: Does Moving Between Nursing Homes Increase Hospitalization and Mortality?

**DOI:** 10.1111/jgs.70554

**Published:** 2026-06-15

**Authors:** Ana T. Montoya, Pil Park, Slim Benloucif, Matthew A. Davis, Julie P. W. Bynum

**Affiliations:** ^1^ Department of Internal Medicine University of Michigan Medical School Ann Arbor Michigan USA; ^2^ Institute for Health Policy and Innovation University of Michigan Ann Arbor Michigan USA; ^3^ Institute of Gerontology University of Michigan Ann Arbor Michigan USA; ^4^ Department of Systems, Populations, and Leadership University of Michigan School of Nursing Ann Arbor Michigan USA; ^5^ Department of Learning Health Sciences University of Michigan Medical School Ann Arbor Michigan USA

**Keywords:** death, hospitalization, nursing home, transfer

## Abstract

**Background:**

Transfer trauma, defined as the negative consequences of moving from one environment to another, can affect some long‐term nursing home (NH) residents who are relocated between facilities. However, it remains unclear whether such transfers are associated with serious acute health risks, such as hospitalization or death, and whether these outcomes should be considered indicators of transfer trauma.

**Methods:**

We conducted a retrospective cohort study to determine whether long‐term NH residents who experience a transfer are at greater risk for hospitalization or death compared to residents who remain in the same facility. We used a 20% national Medicare sample linked to minimum data set (MDS) assessments. Long‐term NH residents who underwent a NH‐to‐NH transfer in 2019 were matched 1:1 with residents from the same facility in the same calendar month who did not transfer. Outcomes were hospitalization and death within 90 days following the transfer. We used conditional logistic regression models, adjusted for demographic variables, dual enrollment status, activities of daily living, cognitive function, and comorbidity scores to assess the association of transfer with outcomes.

**Results:**

A total of 1375 matched resident pairs (*n* = 2750) were analyzed. The number of hospitalizations did not differ between the transfer and non‐transfer groups (173 vs. 160, respectively; *p* = 0.45). Ninety‐day mortality also did not differ between the two groups; however, exact death counts are not reported in accordance with CMS small cell suppression rules. In adjusted analysis, transfer status was not associated with higher odds of 90‐day hospitalization (OR = 1.18 [95% CI: 0.91, 1.53]) or death (OR = 1.58 [95% CI: 0.27, 9.40]).

**Conclusion:**

Among long‐term NH residents, transfer to another NH was not associated with a higher risk of hospitalization or mortality within 90 days post‐transfer. These findings suggest that NH‐to‐NH transfers, when necessary, may not inherently increase severe acute health risks for this population.

## Summary

- Key Points
  ○ Nursing home (NH)‐to‐NH transfer was not associated with increased risk of 90‐day hospitalization or death among long‐stay NH residents.
  ○ Future evaluations of adverse outcomes associated with NH transfers (i.e., transfer trauma) should prioritize changes in residents' health status across multiple domains, rather than focusing solely on severe events such as hospitalization or mortality.
- Why Does This Paper Matter?
  ○ Long‐term NH residents may experience transfer trauma following a relocation to another NH. Close monitoring of residents' health after such transfers is essential for early detection of transfer trauma and the timely implementation of interventions, ultimately contributing to improved quality of life.

## 1 Introduction

For long‐term nursing home (NH) residents, the NH serves as both home and primary caregiving environment. However, many residents are transferred from one NH to another within a year due to facility or unit closures, changes in care needs or insurance coverage, moving closer to family, or other personal preferences [1, 2, 3, 4, 5]. These transfers can disrupt relationships and routines, negatively impacting resident well‐being. The negative consequences associated with transfers have been collectively referred to as “transfer trauma” or “relocation stress syndrome” and encompass a spectrum of outcomes, including functional and cognitive decline, loneliness and isolation, depression, and behavioral changes [3, 4, 5, 6, 7, 8, 9, 10, 11, 12]. Furthermore, there is concern about the heightened risk of serious safety events, hospitalizations, and even death [6, 7, 8, 13, 14, 15, 16, 17].

Previous studies have assessed transfer trauma using one or a limited set of discrete outcomes. In our prior research, we developed a composite measure that integrates multiple clinically relevant submeasures across functional, cognitive, behavioral, and emotional domains, using validated scales within the minimum data set (MDS). This revealed that 20%–30% of long‐term NH residents experienced transfer trauma following a transfer between NHs [18].

However, the association between NH‐to‐NH transfer and severe outcomes like hospitalization and death is unclear. Some studies report increased risks, while others have found no difference or even lower risks [6, 13]. Many of these studies were conducted during events like natural disasters or the COVID‐19 pandemic, limiting generalizability and potentially confounding the impact of transfers.

In this study, we aim to evaluate whether NH‐to‐NH transfer is associated with an increased risk of hospitalization or death. We hypothesized that residents who undergo a transfer will have higher rates of hospitalization and death compared to those who do not. These findings will inform whether to include hospitalization and death in the composite measure of transfer trauma.

## 2 Methods

### 2.1 Data Source and Study Population

This study used a 20% sample of national Medicare administrative data linked to MDS from 2018 to 2020. The MDS contains longitudinal information from federally required assessments in Medicare or Medicaid‐certified NHs. Resident demographics and dual Medicare and Medicaid enrollment were obtained from Medicare Beneficiary Summary File (MBSF); comorbidities from Medicare Provider Analysis and Review (MedPAR) and Medicare Carrier files. Transfer status, index date of transfer, and facility were determined from MDS. Hospitalization data were obtained from MedPAR, and mortality information was sourced from MBSF.

We identified long‐term NH residents in 2019, defining long‐term residents as those with an MDS assessment in 2019 who stayed in a nursing facility for ≥ 100 consecutive days. The University of Michigan Institutional Review Board determined this study to be exempt from review.

### 2.2 NH Transfer

Residents were allocated to the transfer group if they had a direct NH‐to‐NH transfer between January 1–December 31, 2019. A direct NH‐to‐NH transfer was identified if a resident had MDS assessments from two distinct NHs within a 1 day interval, with the discharge assessment indicating discharge to a NH, and the corresponding subsequent assessment indicating admission from a NH. Residents who experienced an intercurrent hospitalization between the NH transfer were not included. The index date was the date of NH‐to‐NH transfer. If multiple transfers occurred within the calendar year, only the first transfer of the year was considered. Residents were excluded if they were younger than 66 years on the index date, if they were enrolled in hospice, or if they lacked continuous Medicare fee‐for‐service enrollment during the 12 months preceding or 3 months following the index transfer date.

To establish the non‐transfer group, residents in the transfer group were matched in a 1:1 ratio with residents who had a MDS assessment in the same calendar month, in the same NH, and did not undergo a NH‐to‐NH transfer. For residents in the non‐transfer group, the index date was defined as the date of the MDS assessment used for matching. The same age and Medicare enrollment exclusion criteria as used for the transfer group were applied. (Figure S1).

### 2.3 Acute Care Hospitalization and Death

The primary outcomes were acute care hospitalization and mortality within 90 days of the index date. Hospitalization was defined by the presence of an acute care hospital claim in the MedPAR file. Hospital length of stay and primary diagnoses for hospitalization were assessed. Date of death was obtained from the MBSF file.

### 2.4 Covariates

Covariates obtained from MDS data included marital status, functional impairment in activities of daily living (ADL), assessed by the long‐form ADL Scale [19]; cognitive impairment as determined by the Cognitive Function Scale (CFS) [20]; health instability using the Changes in Health, End‐stage disease, and Signs and Symptoms (CHESS) score [21]; and the Elixhauser Comorbidity Index score [22]. ADL impairment was categorized using Long‐Form ADL score into: not/mildly impaired (0–13), moderately impaired (14–19), and severely impaired (20–28). Cognitive impairment was categorized using CFS score into: no impairment (1), mild impairment (2), moderate impairment (3), and severe impairment (4). Covariates from Medicare administrative data include resident demographic variables (age, gender, race, and marital status) and dual Medicare and Medicaid enrollment.

### 2.5 Statistical Analyses

We used descriptive statistics to compare long‐term NH residents who had a NH‐to‐NH transfer and those who did not. Differences in means were evaluated using paired *t*‐tests; McNemar's test was used to compare proportions; and Wilcoxon Signed‐Rank test was used to assess differences in medians. Associations between transfer status and the primary outcomes (hospitalization or death) were examined using conditional logistic regression models, adjusted for demographic variables (age, gender, race/ethnicity, marital status), dual Medicare and Medicaid enrollment, ADL functional impairment, cognitive impairment, and comorbidity scores. Statistical analyses were conducted using Stata version 18.0 (StataCorp LLC) and SAS version 9.4 (SAS Institute Inc).

## 3 Results

A total of 1375 long‐term NH residents who underwent a NH‐to‐NH transfer met the inclusion criteria and were matched with 1375 long‐term NH residents who did not transfer. These individuals resided in 1248 NHs. Residents in the transfer group were younger than those in the non‐transfer group (mean age 80.4 [SD 9] vs. 81.2 [SD 9] years, respectively; *p* = 0.01) (Table 1). The groups had similar characteristics: two‐thirds of residents were female, predominantly white, the majority were dually enrolled in Medicare and Medicaid, had similar median Elixhauser Comorbidity Index, median ADL scores, and proportion of comorbidities with one exception. COPD/asthma was more prevalent in the transfer group (36% vs. 32%; *p* = 0.02). The transfer group had a higher mean number of inpatient stays in the previous 12 months than the non‐transfer group (0.6 vs. 0.5; *p* = 0.08).

**TABLE 1:** Characteristics of long‐term nursing home residents by transfer status, 2019.

	Transfer *n* = 1,375	Non‐transfer *n* = 1,375	*p* ^a^
Mean age in years (SD)	80.4 (9)	81.2 (9)	0.01
Age category, *n* (%)			0.01
Age 65–69	187 (14)	169 (12)	
Age 70–74	215 (16)	202 (15)	
Age 75–79	240 (18)	222 (16)	
Age 80–84	252 (18)	250 (18)	
Age 85–89	247 (18)	249 (18)	
Age 90+	234 (17)	283 (21)	
Gender, *n* (%)			0.967
Female	908 (66)	907 (66)	
Male	467 (34)	468 (34)	
Race/ethnicity, *n* (%)			0.890
Non‐Hispanic White	1089 (79)	1085 (79)	
Non‐Hispanic Black	180 (13)	180 (13)	
Hispanic	66 (5)	73 (5)	
Other	40 (3)	37 (3)	
Marital status, *n* (%)			0.009
Married	296 (22)	242 (18)	
Not married^b^	1079 (79)	1133 (82)	
Dual enrollment, *n* (%)	1201 (87)	1217 (89)	0.349
Median ADL score (IQR)	17 (10, 20)	17 (11, 20)	1.00
ADL impairment category, *n* (%)			0.135
Not/mildly impaired (0–13)	480 (35)	451 (33)	
Moderately impaired (14–19)	527 (38)	527 (38)	
Severely impaired (20‐28)	368 (27)	397 (29)	
Cognitive impairment category^c^ *n* (%)			0.222
No impairment	467 (34)	429 (31)	
Mild impairment	338 (25)	332 (24)	
Moderate impairment	419 (31)	484 (35)	
Severe impairment	151 (11)	130 (10)	
Comorbidities^d^ *n* (%)			
Hypertension	1252 (91)	1224 (90)	0.071
Congestive heart failure	482 (35)	470 (34)	0.622
COPD/asthma	500 (36)	441 (32)	0.015
Diabetes	658 (48)	655 (48)	0.907
Chronic kidney disease	436 (32)	442 (32)	0.805
Median Elixhauser Comorbidity Index score^d^ (IQR)	6 (4, 9)	6 (4, 8)	0.103
CHESS category, *n* (%)			0.183
Lower health instability (0–2)	1324 (96)	1310 (95)	
Higher health instability (3–5)	51 (4)	65 (5)	
Mean number of inpatient stays (SD)	0.6 (1)	0.5 (1)	0.082

Abbreviations: ADL, activities of daily living; CHESS, changes in health, end‐stage disease, and symptoms and signs; COPD, chronic obstructive pulmonary disease; IQR, interquartile range; SD, standard deviation.

^a^ Paired *t*‐test used to compare differences in the mean; McNemar's test used to compare proportions; Wilcoxon Signed‐Rank test used to evaluate differences in medians.

^b^ Not married includes never married, widowed, separated, divorced, or missing.

^c^ Cognitive impairment categories by Cognitive Function Scale (CFS) score.

^d^ Identified in the 12‐month look‐back period.

### 3.1 NH Transfer and Acute Care Hospitalization

In unadjusted analyses, the percentage of residents who had a hospitalization within 90 days of the index date did not differ between the transfer and non‐transfer groups (Figure 1). Among the 1375 residents who had a NH‐to‐NH transfer, 173 (12.6%) had a hospitalization within 90 days of transfer compared to 160 (11.6%) among those who did not have a NH‐to‐NH transfer, *p* = 0.45. Of the 173 residents who had a hospitalization within 90 days of transfer, the majority (141) had one hospitalization, and 18% (32) had two or more. Similarly, of the 160 residents who did not have a NH‐to‐NH transfer, the majority (130) had one hospitalization, and 19% (30) had two or more. Hospital length of stay varied from 1 to 135 days, and the mean length of stay did not differ between the two groups. The three most common primary diagnoses for hospitalization in both the transfer and non‐transfer groups were: sepsis, urinary tract infection, and pneumonia.

**FIGURE 1:**
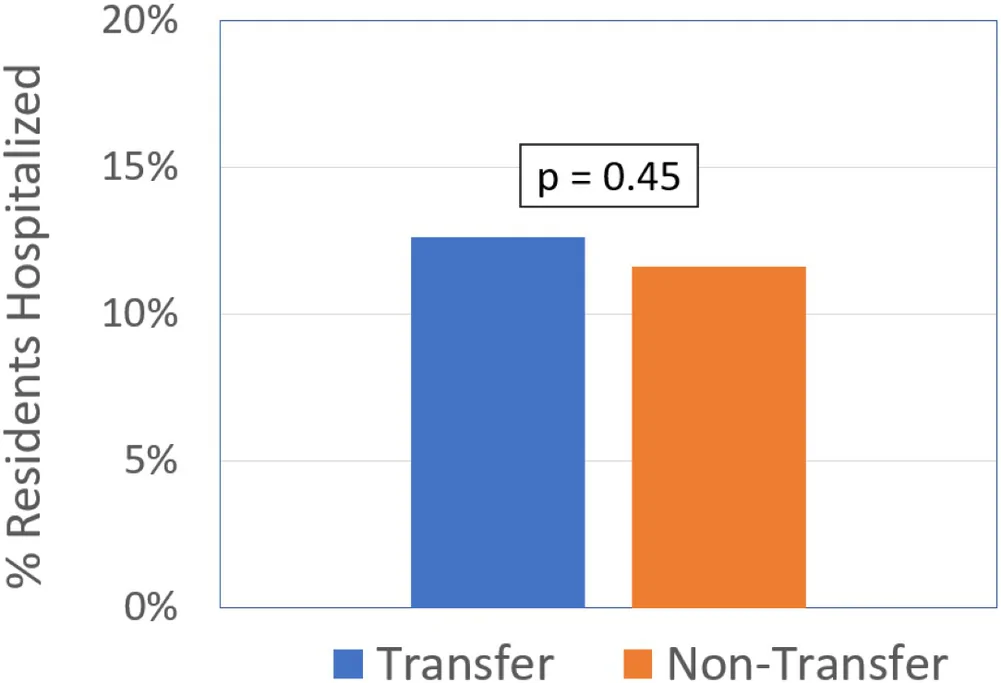
90‐day hospitalization among long‐term nursing home residents by transfer status. Statistical significance was assessed with the Wilcoxon signed‐rank test, and corresponding *p* value is reported.

In adjusted analyses, no statistically significant association between transfer status and acute care hospitalization (OR 1.18 [95% CI: 0.91, 1.53]) was observed. Medicare and Medicaid dual enrollment and severe cognitive impairment were associated with lower odds of hospitalization (OR 0.35 [95% CI: 0.18, 0.71], OR 0.43 [95% CI: 0.21, 0.88]; respectively), while severe ADL impairment and a higher Elixhauser Comorbidity Index Score were associated with higher odds of hospitalization after transfer (OR 1.72 [95% CI: 1.02, 2.92], OR 1.19 [95% CI: 1.12, 1.27]; respectively) (Table 2).

**TABLE 2:** Adjusted odds ratios for the association between transfer status and 90‐day hospitalization and death.

	Hospitalization OR (95% CI)	Death OR (95% CI)
Transfer (ref: non‐transfer)	1.18 (0.91, 1.53)	1.58 (0.27, 9.40)
Age in years	1.00 (0.97, 1.02)	0.96 (0.81, 1.15)
Gender (ref: male)		
Female	1.04 (0.71, 1.52)	1.89 (0.18, 19.76)
Race/ethnicity (ref: non‐Hispanic White)		
All others	1.32 (0.79, 2.22)	0.38 (0.02, 9.47)
Marital status (ref: married)		
Not married^a^	1.43 (0.86, 2.40)	0.66 (0.03, 13.32)
Dual enrollment	**0.35 (0.18, 0.71)**	0.43 (0.01, 18.03)
ADL impairment category^b^ (ref: not/mildly impaired)		
Moderately impaired	1.18 (0.73, 1.88)	1.40 (0.06, 34.89)
Severely impaired	**1.72 (1.02, 2.92)**	1.04 (0.03, 38.07)
Cognitive impairment category^c^ (ref: no impairment)		
Mild impairment	0.77 (0.47, 1.28)	5.19 (0.10, 274.43)
Moderate impairment	0.61 (0.36, 1.03)	0.99 (0.10, 9.91)
Severe impairment	**0.43 (0.21, 0.88)**	2.54 (0.14, 45.73)
Elixhauser Comorbidity Index Score^d^	**1.19 (1.12, 1.27)**	1.24 (0.78, 1.98)

*Note:* Bold value defines statistically significant difference.

Abbreviations: ADL, activities of daily living; CI, confidence interval; OR, odds ratio.

^a^ Not married includes never married, widowed, separated, divorced, or missing.

^b^ ADL impairment categories by Long‐Form ADL Score: not/mildly impaired (0–13), moderately impaired (14–19), severely impaired (20–28).

^c^ Cognitive impairment categories by Cognitive Function Scale (CFS) score: no impairment (1), mild impairment (2), moderate impairment (3), severe impairment (4).

^d^ Identified in the 12‐month look‐back period.

### 3.2 NH Transfer and Mortality

In unadjusted analyses, the percentage of residents who died did not differ between the transfer and non‐transfer groups; however, exact death counts in each group are too low (< 11) to be reported in accordance with CMS small cell suppression rules.

In adjusted analyses, transfer status was not associated with higher odds of death (OR 1.58 [95% CI: 0.27, 9.40]) (Table 2).

## 4 Discussion

In this national cohort study of long‐term NH residents, we found that transfers between NHs did not significantly affect the odds of hospitalization or death within 90 days. These results suggest that necessary relocations between NHs may not increase the risk of severe short‐term health outcomes for this population. Such findings may provide reassurance to clinicians, residents, and families navigating these complex transitions and help to guide clinical practice, inform policy, and support decision‐making regarding transitions in NHs.

Our findings are notable in the context of previous research highlighting the potential risks associated with NH transfers. In our cohort, 12%–13% of long‐term NH residents were hospitalized, regardless of whether they underwent a NH‐to‐NH transfer. This rate is lower than prior reports, which show hospitalization rates of 18 to 37% measured at various time intervals [17, 23, 24, 25] and 12% to 21% following transfers [8]. Many of these studies focused on specific populations who may have higher risk of subsequent hospitalization, such as residents with severe functional impairment, or those newly admitted to NHs. In contrast, our cohort encompassed all long‐term NH residents residing in the facility during the study period, representing a range of functional status levels and variable NH lengths of stay.

Overall mortality among long‐term NH residents in our study was under 1% within 90 days of the index date, irrespective of experiencing a NH‐to‐NH transfer—a rate lower than previous reports of 7% to 18% for residents with severe functional impairment [7, 8]. Mortality of 6% and 24% have been reported following NH‐to‐NH transfers related to disaster evacuations—situations where increased mortality may be attributable to limited resources and conditions during the disaster, rather than the transfer process itself [8, 9, 13, 26, 27]. A previous review showed mixed findings on mortality and suggested that NH‐to‐NH transfer may not be associated with elevated mortality [6]. It is important to note that residents enrolled in hospice were excluded in this study. Our study confirms the lack of association between transfer and death, however, the low death rate limits our power to detect a difference.

Our study has several limitations. Our analyses were conducted on a 20% national sample of Medicare fee‐for‐service beneficiaries, which may limit generalizability to the Medicare advantage enrollees. The low numbers of residents in each specific minority racial/ethnic group necessitated combining them into a single category, obscuring any potential between‐group differences. As an observational study, residual confounding cannot be ruled out, and relationships may not be causal in nature. Previous research has linked higher rates of hospitalization and mortality to institutional factors, such as for‐profit status and staffing variations [25, 28]. As resident pairs were matched within the same NH, we could not study facility characteristics. Finally, the reasons for NH‐to‐NH transfer are not captured in the MDS or Medicare claims data and therefore we cannot address how specific reasons for transfer may differentially impact resident outcomes.

To comprehensively study transfer trauma, it is crucial to employ a measurement tool that captures multiple domains of potential change in the resident's condition, rather than relying solely on isolated outcomes which have been used in many prior studies. Given the broad range of adverse outcomes associated with transfer, we previously developed a composite measure to capture the multiple domains of transfer trauma, including functional and cognitive outcomes, and also behavioral, emotional, and other indicators. In that measure, we initially included hospitalization and death aligned with some prior studies; however, we noted challenges with their inclusion. The main concern was the influence of contextual factors highlighted by the COVID pandemic. In our first study, transfers may have been triggered by COVID infection, which then confounded the relationship between transfer and hospitalization or death. In addition, outcomes outside the NH (such as death after hospitalization) were unobservable. In the present study, utilizing better data to capture hospitalization and death accurately and different time period, we found that these outcomes do not appear to be associated with NH‐to‐NH transfer. Consequently, we recommend that future use of the transfer trauma composite measure not include hospitalization or death as components.

In conclusion, our study suggests that NH‐to‐NH transfers do not inherently increase the risk for short‐term hospitalization or mortality among long‐term residents, and such outcomes should not routinely be used as indicators of transfer trauma. While ongoing efforts to improve communication, continuity of care, and support for residents and families during transitions remain essential, our findings suggest that necessary transfers may not inherently increase severe acute health risks for this population.

## Author Contributions

Study concept and design: Ana T. Montoya, Matthew A. Davis, and Julie P.W. Bynum. Acquisition of data: Ana T. Montoya, Pil Park, and Slim Benloucif. Analysis and interpretation of data: All authors. Preparation of manuscript: All authors.

## Funding

This work was supported by Claude D. Pepper Older Americans Independence Center, University of Michigan, (P30AG024824).

## Disclosure

This work was funded by the Claude D. Pepper Older Americans Independence Center (P30AG024824). The sponsor had no role in the design, methods, data collection, analysis, or preparation of the paper.

## Conflicts of Interest

The authors declare no conflicts of interest.

## Supporting information


**Figure S1:** Flow diagram for the identification of long‐term nursing home (NH) residents who undergo a NH‐to‐NH transfer.
