# Supplementary material for: Transfer Trauma: Does Moving Between Nursing Homes Increase Hospitalization and Mortality?
Source: J Am Geriatr Soc. 2026 Jun 15;74(10):3025–31. doi: 10.1111/jgs.70554 (PMC13723218; doi:10.1111/jgs.70554)
Supplement: Supplementary file 1 [file JGS-74-3025-s001.pdf]

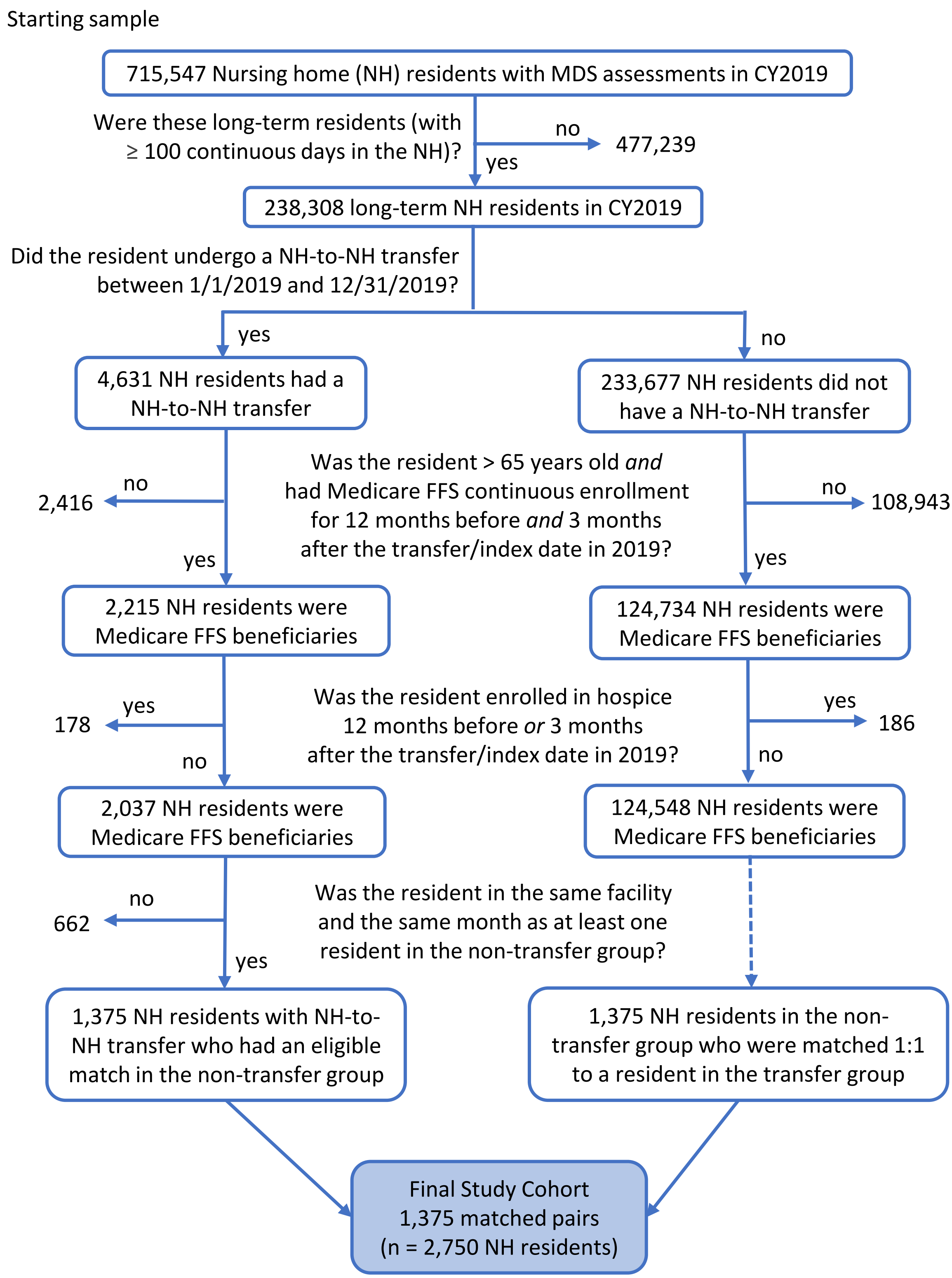

Starting sample
715,547 Nursing home (NH) residents with MDS assessments in CY2019
Were these long-term residents (with ≥ 100 continuous days in the NH)?
no
477,239
yes
238,308 long-term NH residents in CY2019
Did the resident undergo a NH-to-NH transfer between 1/1/2019 and 12/31/2019?
yes
no
4,631 NH residents had a NH-to-NH transfer
233,677 NH residents did not have a NH-to-NH transfer
Was the resident > 65 years old *and* had Medicare FFS continuous enrollment for 12 months before *and* 3 months after the transfer/index date in 2019?
no
2,416
yes
no
108,943
yes
2,215 NH residents were Medicare FFS beneficiaries
124,734 NH residents were Medicare FFS beneficiaries
Was the resident enrolled in hospice 12 months before *or* 3 months after the transfer/index date in 2019?
yes
178
no
yes
186
no
2,037 NH residents were Medicare FFS beneficiaries
124,548 NH residents were Medicare FFS beneficiaries
Was the resident in the same facility and the same month as at least one resident in the non-transfer group?
no
662
yes
1,375 NH residents with NH-to-NH transfer who had an eligible match in the non-transfer group
1,375 NH residents in the non-transfer group who were matched 1:1 to a resident in the transfer group
Final Study Cohort
1,375 matched pairs
(n = 2,750 NH residents)
